# Decompression illness with hypovolemic shock and neurological failure symptoms after two risky dives: a case report

**DOI:** 10.14814/phy2.13094

**Published:** 2017-03-22

**Authors:** Sebastian Klapa, Johannes Meyne, Wataru Kähler, Frauke Tillmans, Henning Werr, Andreas Binder, Andreas Koch

**Affiliations:** ^1^ Section for Maritime MedicineInstitute of Experimental MedicineChristian‐Albrechts‐University Kielc/o German Naval Medical InstituteKopperpahler Allee 120Kronshagen24119Germany; ^2^ Department of NeurologyUKSH Campus KielSchittenhelmstraße 10Kiel24105Germany

**Keywords:** Decompression illness, hypovolemic shock syndrome, patent foramen ovale, systemic inflammatory response syndrome

## Abstract

Hypovolemia is known to be a predisposing factor of decompression illness (DCI) while diving. The typical clinically impressive neurological symptoms of DCI may distract from other symptoms such as an incipient hypovolemic shock. We report the case of a 61‐year‐old male Caucasian, who presented with an increasing central and peripheral neural failure syndrome and massive hypovolemia after two risky dives. Computed tomography (CT) scans of the chest and Magnetic resonance imaging scans of the head revealed multiple cerebral and pulmonary thromboembolisms. Transesophageal echocardiography showed a patent foramen ovale (PFO). Furthermore, the patient displayed hypotension as well as prerenal acute kidney injury with elevated levels of creatinine and reduced renal clearance, indicating a hypovolemic shock. Early hyperbaric oxygen (HBO) therapy reduced the neurological deficits. After volume expansion of 11 liters of electrolyte solution (1000 mL/h) the cardiopulmonary and renal function normalized. Hypovolemia increases the risk of DCI during diving and that of hypovolemic shock. Early HBO therapy and fluid replacement is crucial for a favorable outcome.

## Introduction

Divers are known to develop hypovolemia due to immersion‐induced diuresis and loss of fluids through respiration. As a result of concomitant reduction in plasma there is a detectable relative increase in hemoglobin (Hb), hematocrit (Hct), and serum proteins (Edmonds et al. 2012a). Furthermore, reduced blood fluids may negatively affect both blood viscosity and dynamic blood flow. A reduction in tissue blood perfusion rate with impaired nitrogen clearance may promote the development of decompression illness (DCI) (Boussuges et al. 1996; Newton et al. 2008; Edmonds et al. 2012a). Common symptoms of DCI include cutaneous abnormalities (rash, marbling, and swelling), joint, and muscle pain. Moreover, neurological symptoms can be identified in nearly half (49.4%) of the patients ranging from mild paresis to hemiplegia, paraplegia, and coma. Cardiopulmonary symptoms seem to occur less frequently (12.7%) (Xu et al. 2012).

## Case Report

**Figure 1:**
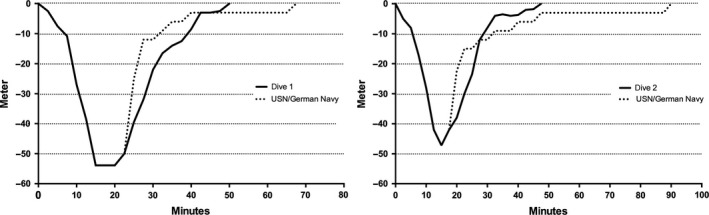
Dive profiles (dive 1 left, dive 2 right) in comparison to US Navy/German Navy standards. Both dives made several decompression stages necessary, which the patient had not completely fulfilled. Furthermore, surface time of 76 min between the dives was inadequate short.

We present a 61‐year‐old experienced male Caucasian diver with more than 4000 uncomplicated dives. He primarily presented with fatigue, vertigo, persistent nystagmus, diplopia and a progressive loss in motor strength of the lower extremities, 4 h after completion of two strenuous dives to 54 m and 48 m using regular compressed air. Both dives required decompression stops according to the standards of the United States Navy (USN) and German Navy, which were partially omitted by the patient (Fig. 1).

In addition, the surface time of 76 min between the two dives was inadequate with respect to these standards. The fluid consumption consisted of 250 mL of a hypertonic soft drink before the first dive and 330 mL of the same drink between dives. 500 mL of water were consumed after both dives. Pre‐existing conditions as well as infections were denied. Environmental conditions were adequate for diving (ambient temperature at 20°C, water temperature of the lake at 10°C/4°C below the 12 m‐thermocline). Technical problems and an emergency ascent were excluded. Three hours after the last dive and the onset of symptoms, the diver was transported to our hospital by the emergency service.

On general examination he looked sweaty and was markedly pale. Primary survey confirmed a Glasgow Coma Score of 15, heart rate of 120/min, blood pressure of 75/50 mmHg, and tympanic temperature of 35.4°C with oxygen saturations of 100% on noninvasive ventilation of 100% oxygen. On neurological examination he presented a right‐directed spontaneous horizontal nystagmus and paraparesis with positive pyramidal tract signs. Initially, hematocrit was significantly increased to 65% consistent with hemoglobin of 22.1 g/dL and leukocytosis of 40.45 × 10^9^/L. Serum levels of creatinine (165 *μ*mol/L), potassium (4.70 mol/L), procalcitonin (13.22 *μ*g/L), lactate (5.5 mmol/L), and C‐reactive protein (72.9 mg/L) were elevated (Table 1).

**Table 1:** Timeline of radiological and laboratory investigations: pathological laboratory values are highlighted in bold

Timeline (h)	4	6	8	18	24	48	72	96	120	144	168
HBO		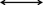		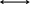		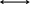	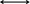	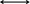	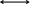		
CT–scans chest		X		X							
MRI – scans head						X					
Hematocrit in % (39.5–50.5)	**65**	**55.8**			39.4			30.5	30.9	31.2	30.2
Hemoglobin in g/dl (13.5–17.2)	**22.1**	**19.6**			13.6			10.3	10.6	10.7	10.8
Leukocytes × 10^9^/L (3.9–10.2)	**40.45**	**37.34**			**22.69**			12.11	10.44	8.46	10.3
Creatinine in *μ*mol/L (59–104)	**165**				**143**			82	72	74	
Potassium in mol/L (3.50–4.50)	**4.70**	**5.04**			4.36			4.03	3.17	3.76	3.71
Procalcitonin in *μ*g/L (<0.05)	**13.22**				**4.87**			**2.01**			
Lactate in mmol/L (0.5–2.2)	**5.5**	**4.4**			1.7						
C‐reactive protein in mg/L (<5.0)	**72.9**				**52**			14.8	8.24	5.65	5.39

CT, computed tomography; MRI, Magnetic resonance imaging; HBO, hyperbaric oxygen

**Figure 2:**
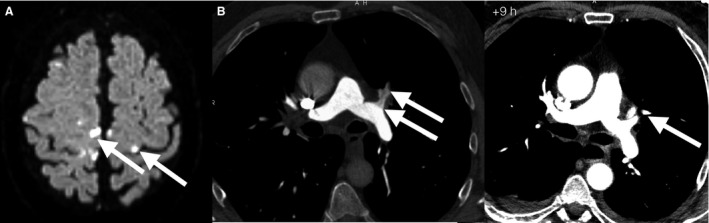
(A) Magnetic resonance imaging scan of the head (diffusion‐weighted imaging) multiple cerebral thromboembolisms according to the central region, superior, and middle frontal gyrus. (B) CT scan of the chest 6 h after the first symptoms: multiple pulmonary thromboembolisms of the segmental arteries. Follow‐up CT scans of the chest 9 h later: no pulmonary thromboembolism of the same segmental arteries. CT, computed tomography; DWI, diffusion‐weighted imaging.

Diagnosis of decompression illness with neurological symptoms as well as a severe hypovolemic shock syndrome was made. CT scans of the chest showed pulmonary thromboembolisms, visible gas bubbles could not be identified (Fig. 2). However, there were no clinical signs of deep vein thrombosis of the limbs or of generalized edema. A transthoracic echocardiogram displayed age‐appropriate normal findings, in particular, no signs for pulmonary arterial hypertension or right‐heart failure. After stabilization of the vital parameters with catecholamines (norepinephrine 0.14 mg/h) for peripheral vasoconstriction and forced intravenous volume expansion (1.000 mL/h isotonic crystalloid maintenance, Ringer's lactate solution) on the intensive care unit (ICU), the patient was transferred to the hyperbaric pressure chamber of the German Naval Medical Institute. The first hyperbaric oxygenation (HBO) started 7 h after the first symptoms using the United States Navy (USN) treatment table 6 with 2 extensions (280 kPa for 80 min followed by 190 kPa for 210 min, US Navy Department 2008). After the first session the patient immediately declared a slight subjective improvement concerning the physical strength of the lower extremities and movement capability. Diplopia was not reported anymore, although the nystagmus was still detectable. 24 h after starting treatment including volume expansion with a cumulative isotonic crystalloid fluid replacement of overall 11 liters circulatory parameters normalized, and dosage of catecholamines could be stopped after 15 h. MRI scans of the head showed multiple cerebral thromboembolisms (Fig. 2). Follow‐up CT scans of the chest 9 h later no longer displayed radiological signs of pulmonary thromboembolisms (Fig. 2).

Values for hematocrit, hemoglobin, potassium, and lactate reverted to normal levels within this time. Serum levels of creatinine, procalcitonin, and C‐reactive protein as well as leukocytosis normalized 4 days later (Table 1).

After additional 5 days of standard HBO sessions according to the USN treatment table 9 (240 kPa for 130 min) (US Navy Department 2008) HBO therapy was stopped. At that time neurological symptoms had significantly improved, but showed no further reduction under HBO therapy.

Two days after the first symptoms, a transesophageal contrast echocardiography revealed a patent foramen ovale (PFO) with spontaneous large right‐to‐left shunt during the valsalva maneuver, which is technically difficult to detect by transthoracic echocardiogram. 16 days after the diving accident the patient had further improved but still presented with a slight unsteadiness and was therefore transferred to a rehabilitation unit.

## Discussion

Looking at the growing popularity of noncommercial SCUBA diving, accidents among recreational divers become increasingly frequent and are potentially life‐threatening (Andersen et al. 2013). However, DCI is still a rare event, with an incidence rate estimated between 0.02% and 0.03% per dive. The risk of DCI increases with age and body‐mass index (Vann et al. 2011)**.** Although some details in the pathomechanism of DCI are still under discussion, two distinct major mechanisms are accepted to be responsible for DCI: On one hand, arterial gas embolism (AGE) can be the consequence of a pulmonary barotrauma due to an acute increase in pressure within the lungs. On the other hand, an excess amount of inert gas, which is liberated over time from saturated tissues can form bubbles in venous blood. This may cause pulmonary as well as secondary arterial embolisms after right‐to‐left shunting. Too fast ascents as well as omitted decompression are common causes for this. Primary or secondary deficit in body water with reduced plasma‐volume and changes in microcirculation are discussed to contribute to reduced inert gas clearance (Boussuges et al. 1996; Newton et al. 2008; Edmonds et al. 2012a; Wilmshurst 2015). Hypovolemia is well known in diving due to immersion‐induced diuresis, and also caused by cold and extremely dry breathing gas (Edmonds et al. 2012a). Changes in hematocrit level ≥48% correlate with persistent neurological sequelae after DCI (Boussuges et al. 1996). In the case presented, profiles of both dives were strenuous, beyond recommendations for recreational diving, and omitted decompression. Our patient consumed only 330 mL of a hypertonic soft drink during the 170 min of both dives including the surface interval, and thus did not maintain an adequate hydration level. Therefore, we consider the initial high hematocrit of 65% and hemoglobin of 22.1 g/dl mainly to be the consequence of massive volume depletion. Additionally, a postdecompression systemic capillary leak syndrome caused by massive gas emboli formation may have contributed to further hemoconcentration. This has recently been discussed in cases, where patients presented with elevated hematocrit, reduced levels of serum albumin, clinical signs of hypotension, and generalized edema (Hills and James 1991; Gempp et al. 2013). While hypoalbuminemia suggests the occurrence of neurological symptoms in DCI (Gempp et al. 2014) our patient showed no signs of generalized edema, although serum albumin was reduced to 30.7 g/L on the 3rd day (in ICU). Considering the rapid therapeutic improvement, we did not give additional albumin or fresh‐frozen plasma. The development of a postdecompression systemic capillary leak syndrome might be linked to a systemic inflammatory response syndrome (SIRS), caused by an excessive immune reaction, and initiated by vascular gas formation. Current data suggest that development of serious DCI might activate the immune system similar to an immune response against spreading infection, exhibiting comparable clinical signs. Our patient presented elevated serum levels of inflammatory markers, such as C‐reactive protein and procalcitonin, and massive leukocytosis of 40.45x10^9^/l, thus meeting 3 of 4 criteria for SIRS (Dellinger et al. 2013): Temperature <36°C, heart rate >90beats/minute and leukocytosis >12 × 10^9^/L. Yet he showed no clinical signs of infection. After venous gas bubble formation, right‐to‐left shunting through a PFO or functionally open intrapulmonary arteriovenous anastomosis (IPAVA) increases the risk of neurological DCI in divers (Vann et al. 2011; Gempp et al. 2012b; Madden et al. 2015). A PFO is a normal variant in about one quarter of adults (Hagen et al. 1984; Andersen et al. 2013), which greatly increases the risk for neurological symptoms in DCI (Lairez et al. 2009). Postaccidental transesophageal echocardiography diagnosed spontaneous shunting and previously unknown PFO in our patient. Though transient pulmonary thromboembolism was assured by CT scan, it remains unclear whether a large PFO was permanently present beforehand or whether a functionally nearly closed PFO reopened due to short‐term acute increased pulmonary pressure. Although no cases of aggravated PFO have been described so far, it is a conceivable mechanism. As it has been discussed that right‐to‐left shunting effects may increase due to exertion in divers (Wilmshurst et al. 1994), it could be assumed that during the two strenuous dives or during pulmonary gas embolization a small PFO was enlarged by changes in pressure, which led to secondary arterialization. Furthermore, this mechanism might explain the lack of radiological signs of earlier possibly diving‐associated cerebral embolisms in the patients MRI. The central neurological symptoms fatigue, vertigo, and diplopia and the peripheral symptom paraparesis were reduced after volume expansion and HBO. It can be assumed that these neurological symptoms were caused by both, DCI and hypovolemic shock. To our knowledge, the presented case of a severe pulmonary and neurologic DCI in an experienced diver is the first documented incident related to such volume depletion resulting in a hypovolemic shock syndrome requiring catecholamines and an aggressive intravenous volume expansion therapy. Although no emergency ascent was performed, the cumulation of the following risk factors contributed to the development of thromboembolisms in lungs and brain: the dives were strenuous and beyond recommendations for recreational diving, the age of the diver, the divers unawareness of having a PFO, and the massive hypovolemia delaying regular clearance of inert gas. Treatment of an acute DCI should include effective on‐site management with initial normobaric 100% oxygen breathing and administration of oral/venous fluids, as well as rapid hyperbaric oxygenation in a pressure chamber in order to terminate further bubble development, reduce size of existing bubbles, and dramatically increase inert gas clearance from tissue and blood (Jüttner et al. 2015). Since fast inert gas clearance from tissues also needs adequate intra‐ and extravascular fluids as transport solvent to the lung, an appropriate volume substitution should be rated highly in every DCI treatment.

## Consent Section

Written informed consent was obtained from the patient for publication of this case report and any accompanying images. A copy of written consent is available for review by the Editor‐in‐Chief of this journal.

## Conflict of Interest

The authors declare that they have no competing interests.
